# Self-Supervised Electroencephalogram Representation Learning for Automatic Sleep Staging: Model Development and Evaluation Study

**DOI:** 10.2196/46769

**Published:** 2023-07-26

**Authors:** Chaoqi Yang, Cao Xiao, M Brandon Westover, Jimeng Sun

**Affiliations:** 1 Computer Science Department, Carle's Illinois College of Medicine, University of Illinois, Urbana Champaign, Urbana, IL, United States; 2 Relativity Inc, Chicago, IL, United States; 3 Harvard Medical School, Boston, MA, United States

**Keywords:** physiological signals, electroencephalogram, EEG, sleep staging, sleep, predict, wearable devices, wearable, self-supervised learning, digital health, mHealth, mobile health, healthcare, health care, machine learning

## Abstract

**Background:**

Deep learning models have shown great success in automating tasks in sleep medicine by learning from carefully annotated electroencephalogram (EEG) data. However, effectively using a large amount of raw EEG data remains a challenge.

**Objective:**

In this study, we aim to learn robust vector representations from massive unlabeled EEG signals, such that the learned vectorized features (1) are expressive enough to replace the raw signals in the sleep staging task, and (2) provide better predictive performance than supervised models in scenarios involving fewer labels and noisy samples.

**Methods:**

We propose a self-supervised model, Contrast with the World Representation (ContraWR), for EEG signal representation learning. Unlike previous models that use a set of negative samples, our model uses global statistics (ie, the average representation) from the data set to distinguish signals associated with different sleep stages. The ContraWR model is evaluated on 3 real-world EEG data sets that include both settings: at-home and in-laboratory EEG recording.

**Results:**

ContraWR outperforms 4 recently reported self-supervised learning methods on the sleep staging task across 3 large EEG data sets. ContraWR also supersedes supervised learning when fewer training labels are available (eg, 4% accuracy improvement when less than 2% of data are labeled on the Sleep EDF data set). Moreover, the model provides informative, representative feature structures in 2D projection.

**Conclusions:**

We show that ContraWR is robust to noise and can provide high-quality EEG representations for downstream prediction tasks. The proposed model can be generalized to other unsupervised physiological signal learning tasks. Future directions include exploring task-specific data augmentations and combining self-supervised methods with supervised methods, building upon the initial success of self-supervised learning reported in this study.

## Introduction

Deep learning models have shown great success in automating tasks in sleep medicine by learning from high-quality labeled electroencephalogram (EEG) data [1]. EEG data are collected from patients wearing clinical sensors, which generate real-time multimodal signal data. A common challenge in classifying physiological signals, including EEG signals, is the lack of enough high-quality labels. This paper introduces a novel self-supervised model that leverages the inherent structure within large, unlabeled, and noisy data sets and produces robust feature representations. These representations can significantly enhance the performance of downstream classification tasks, such as sleep staging, especially in cases where only limited labeled data are available.

Self-supervised learning (specifically, self-supervised contrastive learning) aims at learning a feature encoder that maps input signals into a vector representation using unlabeled data. Self-supervised methods involve two steps: (1) a *pretrain* step to learn the feature encoder without labels and (2) a *supervised* step to evaluate the learned encoder with a small amount of labeled data. During the pretrain step, some recent methods (eg, Momentum Contrast [MoCo] [2] and the simple framework for contrastive learning of visual representations [SimCLR] [3]) use the feature encoder to construct positive and negative pairs from the unlabeled data and then optimize the encoder by pushing positive pairs closer and negative pairs farther away. A positive pair consists of 2 different augmented versions of the same sample (ie, applying 2 data augmentation methods separately to the same sample), while a negative pair is generated from the augmented data of 2 different samples. For example, the augmentation method for EEG data can be denoising or channel flipping. In this practice, existing negative sampling strategies often incur sampling issues [4,5], especially for noisy EEG data, which significantly affects performance [6]. Specifically, in the self-supervised learning setting (without labels), the negative samples are actually random samples, which may be from the same latent class. Using these “negative samples” can potentially undermine model performance.

Technically, this study contributes to the pretrain step, where we address the aforementioned limitations of existing negative sampling strategies (eg, MoCo [2] and SimCLR [3]) by leveraging global data statistics. In contrastive learning, positive pairs provide similarity-related information, while negative pairs provide contrastive information. Both types of information are essential in learning an effective feature encoder. This study proposes a new contrastive learning method, named Contrast with the World Representation (ContraWR). In our ContraWR, we construct positive pairs using data augmentation, similar to existing methods, while we use one global average representation over the data set (called the *world representation*) as the negative sample to provide the contrastive information. Derived from global data statistics, the world representation is robust even in noisy environments, and it follows a new contrastive guidance in the absence of labels: *the representation similarity between positive pairs is stronger than the similarity to the world representation*. Moreover, in this study, we later strengthen our model with an instance-aware world representation for individual samples, where closer samples have larger weights in calculating the global average. Our experiments show that the instance-aware world representation makes the model more accurate, and this conclusion aligns with the findings from a previous paper [6] that harder negative samples are more effective in learning feature encoding.

We evaluated the proposed ContraWR on the sleep staging task with 3 real-world EEG data sets. Our model achieved results comparable to or better than those of recent popular self-supervised methods including MoCo [2], SimCLR [3], Bootstrap Your Own Latent (BYOL) [7], and simple Siamese (SimSiam) [8]. The results also show that self-supervised contrastive methods, especially our ContraWR method, are much more powerful in low-label scenarios than supervised learning (eg, 4% accuracy improvement on sleep staging with less than 2% training data of the Sleep EDF data set).

## Methods

### EEG Data Sets

We considered 3 real-world EEG data sets for this study (the first 2 data sets entirely comprise at-home PSG recordings):

1. The data set of the Sleep Heart Health Study (SHHS) [9,10] is a multicenter cohort study from the National Heart Lung & Blood Institute (Bethesda, Maryland), assembled to study sleep-disordered breathing, which comprises 5804 adult patients older than 40 years and 5445 recordings in the first visit. We used first-visit polysomnography (PSG) data in the experiments. Each recording has 14 PSG channels, and the recording frequency is 125.0 Hz. We used the C3/A2 and C4/A1 EEG channels.
2. The Sleep EDF [11] cassette portion is another benchmark data set collected in a 1987-1991 study of age effects on sleep in healthy Caucasians. The data comprise 78 subjects aged 25-101 years who were taking non–sleep-related medications; the data set contains 153 full-night EEG recordings with a recording frequency of 100.0 Hz. We extracted the Fpz-Cz/Pz-Oz EEG channels as the raw inputs to the model.
3. The Massachusetts General Hospital’s (MGH’s) MGH Sleep data set [1] was collected from MGH’s sleep laboratory, which comprises more than 5000 individuals, where 6 EEG channels (ie, F3-M2, F4-M1, C3-M2, C4-M1, O1-M2, and O2-M1) were used for sleep staging, recorded at a 200.0-Hz frequency. After filtering out mismatched signals and missing labels, we finally curated 6478 recordings.

The data set’s statistics can be found in Table 1, and the class label distribution is shown in Table 2.

### Problem Formulation

To set up the experiments, the raw subject EEG recordings, which are multichannel brain waves, were used. First, the unlabeled subject recordings were grouped as the *pretrain* set, and the labeled recordings were grouped into the *training* or *test* sets. The training and test sets are usually small, but their EEG recordings are labeled, while the pretrain set contains a large number of unlabeled recordings. Within each set, the long recordings are segmented into disjoint 30-second windows. Each window is called *an epoch*, denoted as $x\in R^{C\times N}$. Each epoch has the same format: C input channels and N time stamps from each channel.

For these data sets, the ground truth labels were released by the original data publishers. To align with the problem’s setting, participants were randomly assigned to the pretrain set, training set, and test set in different proportions (90%: 5%: 5% for the Sleep EDF and MGH sets and 98%: 1%: 1% for the SHHS set, since they have different amounts of data). All epochs segmented from a participant are placed within the same set. The pretrain set is used for self-supervised learning; hence, we removed their labels.

In the pretrain step, the EEG self-supervised representation learning problem requires building a feature encoder f(⋅) from the pretrain set (without labels), which maps an epoch x into a vector representation $h\in R^{d}$, where d is the feature dimensionality, such that the representation h can replace raw signals for downstream classification tasks. Evaluation of the encoder f(⋅) was conducted on the training and test data (with labels). We focus on sleep staging as the *supervised* step, where the feature vector of a sample *x* will be mapped to 5 sleep cycle labels, awake (W), rapid eye movement (REM; R), non-REM 1 (N1), non-REM 2 (N2), and non-REM 3 (N3), based on the American Academy of Sleep Medicine’s (AASM’s) scoring standards [12]. Specifically, based on the feature encoder from the pretrain step, the training set is used to learn a linear model on top of the feature vectors, and the test set is used to evaluate the linear classification performance.

### Background and Existing Methods

#### Overview

Self-supervised learning occurs in the pretrain step, and it uses representation similarity to exploit the unlabeled signals, with an encoder network $f(\cdot ):R^{C\times N}\to R^{d}$ and a nonlinear projection network $g(\cdot ):R^{d}\to R^{m}$. Specifically, for a given signal x from the pretrain set, commonly, one applies data augmentation methods a(⋅) to produce 2 different modified signals $x^{\sim \prime }$, $x^{\sim \prime \prime }$ (after this procedure, the format does not change), which are then transformed into $h^{\prime }$, $h^{\prime \prime }\in R^{d}$ by f(⋅) and further into $z^{\prime }$, $z^{\prime \prime }\in R^{m}$ by g(⋅). The vectors $z^{’}$, $z^{’’}$ are finally normalized with the L2 norm onto the unit hypersphere $\frac{z}{\left\|z\right\|}\in S^{m-1}$.

We call $\frac{z^{\prime }}{\left\|z^{\prime }\right\|}$ the *anchor*, $\frac{z^{\prime \prime }}{\left\|z^{\prime \prime }\right\|}$ the *positive sample*, and these 2 together are called a *positive pair*. For the projections $z_{k}$ obtained from other randomly selected signals (by negative sampling strategy), their representation $\frac{z_{k}}{\left\|z_{k}\right\|}$ is commonly conceived of as negative samples (though they are random samples), and any one of them together with the anchor is called a *negative pair* in the existing literature [2,3]. The loss function L is derived from the similarity comparison between positive and negative pairs (eg, encouraging the similarity of positive pairs to be stronger than that of all the negative pairs, referred to as the noise contrastive estimation loss [13]). A common forward flow of self-supervised learning on EEG signals can be illustrated as $x\Rightarrow {}_{a(\cdot )}\;\tilde{x}\Rightarrow {}_{f(\cdot )}\;h\Rightarrow {}_{g(\cdot )}\;z\Rightarrow {}_{L2}\;\frac{z}{\left\|z\right\|}\Rightarrow {}_{loss}\;L$.

For data augmentation, this study used bandpass filtering, noising, channel flipping, and shifting (see the definition in Multimedia Appendix 1 and the visual illustrations in Multimedia Appendix 2). We conducted ablation studies on the augmentation methods in our experiment and have provided the implementation details. To reduce clutter, we also used z to denote the L2 normalized version in the rest of the paper.

#### ContraWR

##### Background

As mentioned above, most existing models use random samples as negative samples, which can introduce issues (that the negative sample might be from the same latent class) for the pretrain step and undermine representation quality. To address the issue, this paper proposes a new self-supervised learning method, ContraWR. ContraWR replaces the large number of negative samples with a single average representation of the batch, called the world representation or global representation. This way is robust as it avoids constructing negative pairs where 2 data are actually obtained from the same latent class. The world representation servers as a reference in our new contrastive principle: the representation similarity between a positive pair should be stronger than the similarity between the anchor and the world representation. Note that the world representation is not fixed but changes with the encoder updating the parameters.

##### The World Representation

Assume z’ is the anchor, z’’ is the positive sample, and $z_{k}$ denotes a random sample. We generate an average representation of the data set, $z_{w}$ as the only negative sample. To formalize, we assume k~p(⋅) is the sample distribution over the data set (ie, k is the sample index), independent of the anchor z’. The world representation $z_{w}$ is defined by $z_{w}=E_{k-p(\cdot )}[z_{k}]$.

Here, we denote $D=[z:\|z\|\leq 1,z\in R^{m}]$. Obviously, $z_{w}\in D$. In the experiment, $z_{w}$ is approximated by the average over each batch; that is, we used the average sample representation over the batch $z_{w}=\frac{1}{M}\sum_{k=1}^{M}z_{k}$ as the world representation, where M is the batch size.

##### Gaussian Kernel Measure

We adopted a Gaussian kernel defined on D, sim(x,y):D×D→(0,1] as a similarity measure. Formally, given 2 feature projections z’, z’’ the similarity is defined as $sim(z^{\prime },z_{k})=exp\left(-\frac{\left\|z^{\prime }z_{k}\right\|}{2\sigma^{2}}\right)$, where σ is a hyperparameter. The Gaussian kernel combined with the following triplet loss gives the alignment and uniformity properties in the loss convergence (Multimedia Appendix 3). When *a* becomes large, the Gaussian kernel measure will reduce to cosine similarity.

##### Loss Function

For the anchor z’, the positive sample z’’ and the world representation $z_{w}$, we devise a triplet loss, $L={\left[sim(z’,z_{w})+\delta -sim(z’,z’’)\right]}_{+}$, where δ>0 is the empirical margin, a hyperparameter. The loss is minimized over batches, ensuring that the similarity of positive pairs sim(z’,z’’), is larger than the similarity to the world representation $sim(z’,z_{w})$, by a margin of δ.

**Figure 1.**
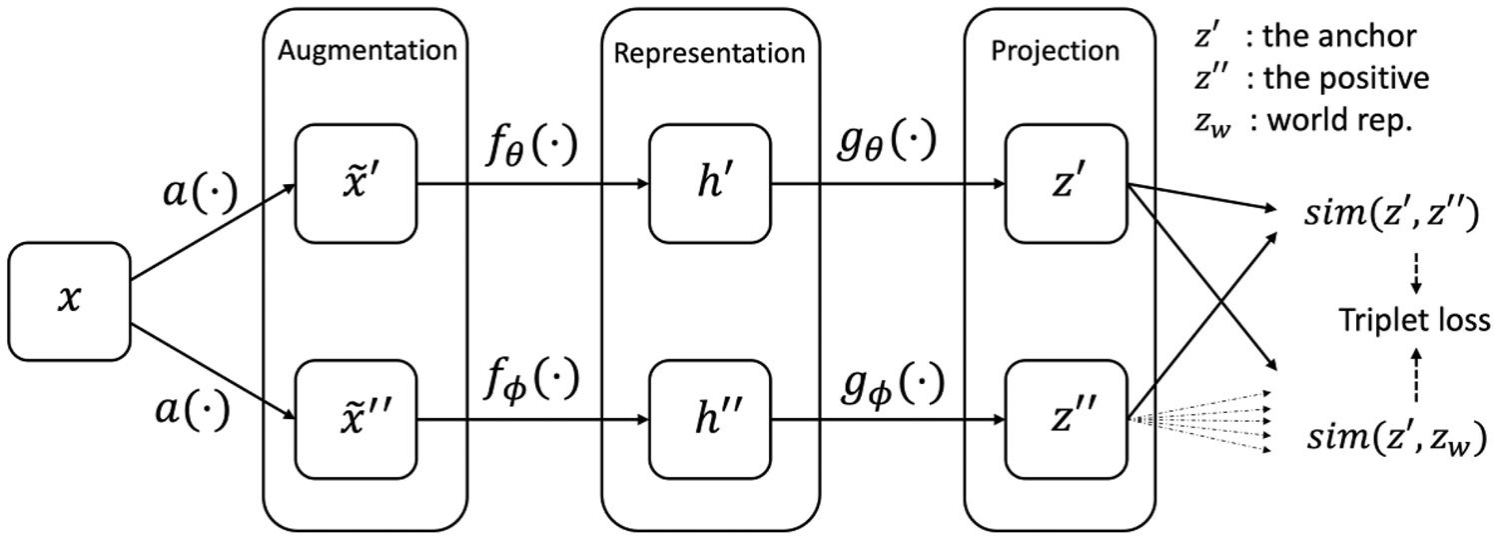
The Contrast with the World Representation (ContraWR) model pipeline. We show the 2-way model pipeline in this figure. The web-based network (upper) is updated by gradient descent, while the target network (lower) is updated by the exponential moving average. Finally, the results of the 2 models form the triplet loss function.

The pipeline of our ContraWR is shown in Figure 1. The online networks $f_{\theta }(\cdot )$, $g_{\theta }(\cdot )$ and the target networks $f_{\varphi }(\cdot )$, $g_{\varphi }(\cdot )$ share an identical network structure. Encoder networks $f_{\theta }(\cdot )$, $f_{\varphi }(\cdot )$ map 2 augmented versions of the same signal to respective feature representations. Then, the projection networks $g_{\theta }(\cdot )$, $g_{\varphi }(\cdot )$ project the feature representations onto a unit hypersphere, where the loss is defined. During optimization, the web-based networks are updated by gradient descent, and the target networks update parameters from the online network with an exponential moving average (EMA) trick [2].

$$\theta^{\left(n+1\right)}\leftarrow \theta^{\left(n\right)}-\eta \cdot \nabla_{\theta }L\;\varphi^{\left(n+1\right)}\leftarrow \lambda \cdot \varphi^{\left(n\right)}+(1-\lambda )\cdot \theta^{\left(n+1\right)}$$

where n indicates the *n*th update, η is the learning rate, and λ is a weight hyperparameter. After this optimization in the pretrain step, the encoder network $f_{\theta }(\cdot )$ is ready to be evaluated on the training and test sets in the supervised step.

#### ContraWR+: Contrast With Instance-Aware World Representation

##### Background

To learn a better representation, we introduced a weighted averaged world representation based on the harder principle: the similarity between a positive pair should be stronger than the similarity between the anchor and the weighted average feature representations of the data set, where the weight is set higher for closer samples. We call the new model ContraWR+. This is a more difficult objective than the simple global average in ContraWR.

##### Instance-Aware World Representation

In this new model, the world representation is enhanced by modifying the sampling distribution to be instance-specific. We define p(⋅∣z) as the instance-aware sampling distribution of an anchor z, which is different from the sample distribution p(⋅) used in ContraWR, $p(\cdot |z)\propto \;\text{exp}\left(\frac{\langle \cdot ,Z\rangle }{T}\right)$, where T>0 is a temperature hyperparameter, such that similar samples are selected with higher probability parametrized by p(⋅∣z). Consequently, for an anchor z’, the instance-aware world representation becomes

$$z_{w}=E_{k∼p\left(\cdot |z^{\prime }\right)}[z_{k}]=\frac{E_{k∼p}\left[\text{exp}\left(\frac{\langle z_{k},z^{\prime }\rangle }{T}\right)\cdot z_{k}\right]}{E_{k∼p}\left[\text{exp}\left(\frac{\langle z_{k},z^{\prime }\rangle }{T}\right)\right]}.$$

Here, T controls the contrastive hardness of the world representation. When T→∞, p(⋅∣z) is asymptotically identical to p(⋅), and the above equation reduces to the simple global average form $z_{w}=E_{k\sim p(\cdot )}[z_{k}]$; while $T\to 0^{+}$, the form becomes trivial, $z_{w}={argmax}_{zk}(sim(z^{\prime },z_{k}))))$. We have tested different T and found that the model is not sensitive to T over a wide range. Here, $z_{w}$ is also practically implemented by using the weighted average over each batch. We can rewrite the similarity measure given the anchor $z_{i}$ and the new world representation $z_{w}$ as:

$$sim(z_{i},z_{w})=sim(z^{\prime },E_{k∼p(\cdot |z^{\prime })}[z_{k}])\;\;=\text{exp}\left({-\frac{1}{2\sigma^{2}}\|z^{\prime }-\frac{E_{k∼p}\left[\text{exp}\left(\frac{\langle z_{k},z^{\prime }\rangle }{T}\right)\cdot z_{k}\right]}{E_{k∼p}\left[\text{exp}\left(\frac{\langle z_{k},z^{\prime }\rangle }{T}\right)\right]}\|}^{2}\right)$$

In this new method, we also used triplet loss as the final objective.

#### Implementations

##### Signal Augmentation

For the experiments, we used four augmentation methods, illustrated in Multimedia Appendix 2: (1) bandpass filtering: to reduce noise, we used an order-1 Butterworth filter (the bandpass is specified in Multimedia Appendix 2); (2) noising: we added extra high- or low-frequency noise to each channel, mimicking the physical distortion; (3) channel flipping: corresponding sensors from the left side and the right of the head were swapped due to symmetricity; and (4) shifting: within one sample, we advanced or delayed the signal for a certain time span. Detailed configurations of augmentation methods vary for the 3 data sets, and we have listed them in Multimedia Appendix 2.

##### Baseline Methods

In the experiments, several recent self-supervised learning methods were implemented for comparison.

MoCo [2] devises 2 parallel encoders with an EMA. It also uses a large memory table to store new negative samples, which are frequently updated.

SimCLR [3] uses an encoder network to generate both anchor and positive samples, where negative samples are collected from the same batch.

BYOL [7] also uses 2 encoders: a web-based network and a target network. They put one more predictive layer on top of the web-based network to predict (reconstruct) the result from the target network, while no negative samples are presented.

SimSiam [8] uses the same encoder networks on 2 sides and also does not use the negative samples.

Average k-nearest neighbor TopX is our developed baseline model, which identifies the top X nearest neighbors for each sample within the batch and uses the average representation of these top X neighbors as the negative sample. We used the same triplet loss as our ContraWR model. In the experiments, we tested X=1, X=5, and X=50. When X approaches the batch size, this model will gradually reduce to ContraWR.

##### Model Architecture

**Figure 2.**
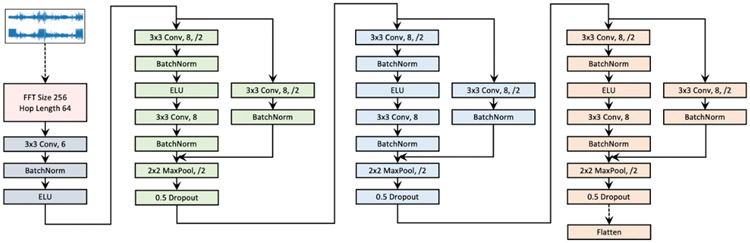
The short-time Fourier transform (STFT) convolutional encoder network. The encoder network first transforms raw signals into spectrogram via STFT, and then a convolutional neural network–based encoder is built on top of the spectrogram. ELU: exponential linear unit; FFT: Fast Fourier Transform; Conv.:convolution operation.

For a fair comparison, all models, including baseline approaches and our models, use the same augmentation and encoder architecture, as shown in Figure 2. This architecture cascades a short-time Fourier transform (STFT) operation, a 2D convolutional neural network layer, and three 2D convolutional blocks. Empirically, we found that the application of neural networks generates better accuracy on the STFT spectrogram of the signals than on the raw signals. The same practices were reported by Yang et al [14,15].

We also considered a supervised model (called *Supervised*) as a reference model, which uses the same encoder architecture and adds a 2-layer fully connected network (128, 256, and 192 units for the Sleep EDF, SHHS, and MGH data sets, respectively) for the sleep staging classification task. The supervised model does not use the pretrain set but is trained from scratch on raw EEG signals in the training set and tested on the test set. We also included an untrained encoder model as a baseline, where the encoder was initialized but not optimized in the pretrain step.

#### Evaluation Protocol

We evaluated performance on the sleep staging task with overall 5-class classification accuracy. Each experiment was conducted with 5 different random seeds. For self-supervised methods, we optimized the encoder for 100 epochs (here, “epoch” is a concept in deep learning) with unlabeled data, used the training set to find a good logistic classifier, and used the test set data for evaluation in accordance with He et al [2] and Chen et al [3]. For the supervised method, we trained the model for 100 epochs on the training set. Our setting ensures the convergence of all models.

## Results

### Better Accuracy in Sleep Staging

Comparisons on the downstream sleep staging task are shown in Table 3.

All self-supervised methods outperformed the untrained encoder model, indicating that the pretrain step does learn some useful features from unlabeled data. We observed that ContraWR and ContraWR+ both outperform the supervised model, suggesting that the feature representations provided by the encoder can better preserve the predictive features and filter out noises than using the raw signals for the sleep staging task, in cases when the amount of labeled data available are not sufficient (eg, less than 2% in Sleep EDF). Compared to other self-supervised methods, our proposed model ContraWR+ also provided better predictive accuracy; that is, about 1.3% on Sleep EDF, 0.8% on SHHS, 1.3% on MGH Sleep. The performance improvements were mostly significant (*P*<.001; comparing MoCo vs Sleep EDF data sets, *P*=.002). MGH Sleep data contain more noise than the other 2 data sets (reflected by the relatively low accuracy with the supervised model on raw signals). Performance gain was notably much more significant on MGH over other self-supervised or supervised models (about 3.3% relative improvement on accuracy), which suggests that the proposed models handle noisy environments better.

### Ablation Study on Data Augmentations

We also inspected the effectiveness of different augmentation methods on EEG signals, shown in Table 4.

We empirically test all possible combinations of 4 considered augmentations: channel flipping, bandpass filtering, noising, and shifting. Since channel flipping cannot be applied by itself, we combined it with other augmentations. The evaluation was conducted on Sleep EDF data with the ContraWR+ model. To sum up, all augmentation methods are beneficial, and collectively, they can further boost the classification performance.

### Varying Amount of Training Data

**Figure 3.**
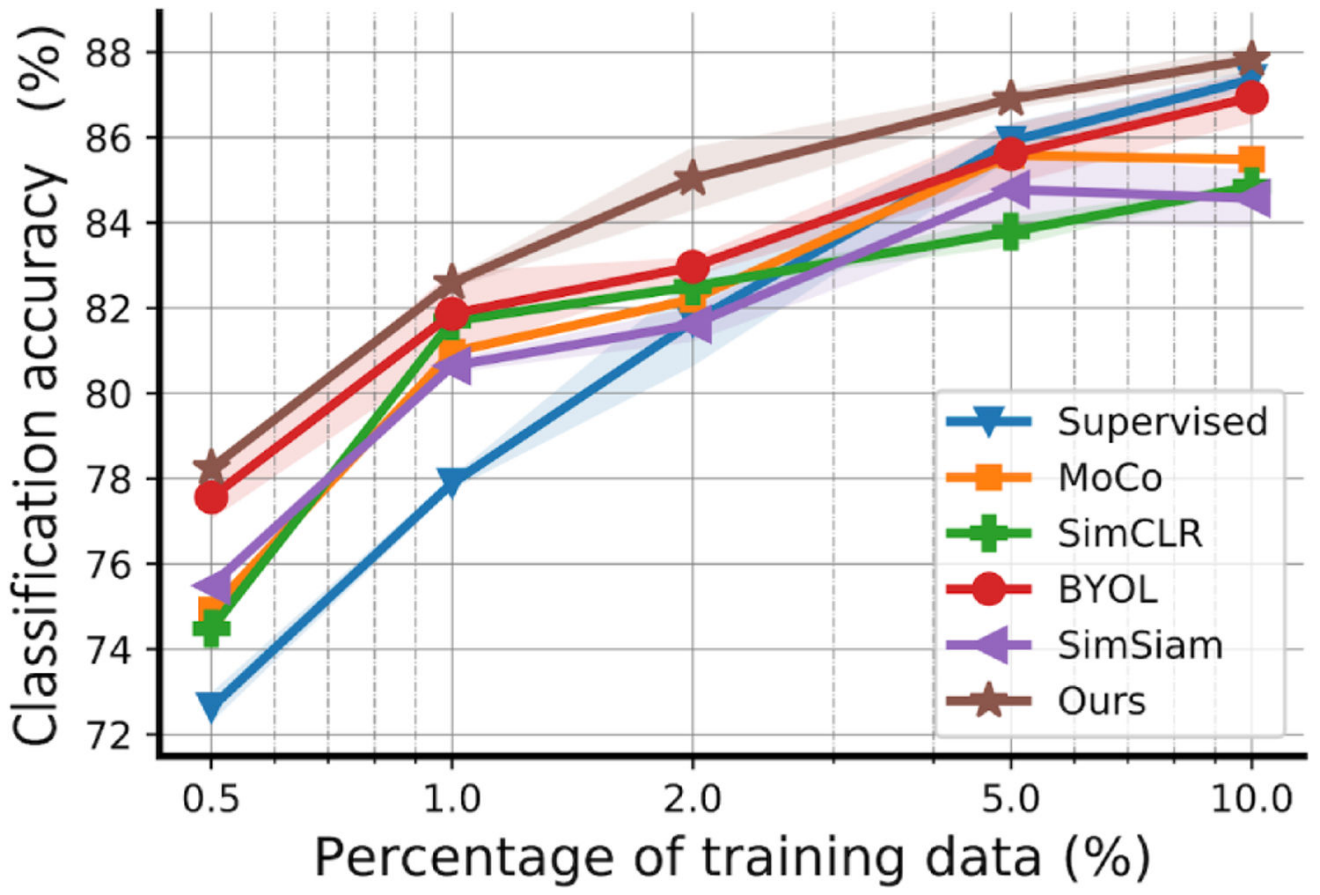
Model performance with different amounts of training data (on the Sleep EDF data set). The curves indicate mean values and shaded areas show the SD of the training/test over 5 random seeds. All models have the same encoder network architecture. For the self-supervised method, we trained a logistic regression model on top of the frozen encoder with the training set, and for the supervised model, we trained the encoder along with the final nonlinear classification layer from scratch with the training set. The proportion of training data is 0.5%, 1%, 2%, 5%, and 10%. Each configuration runs with 5 different random seeds and the error bars indicate the SD over 5 seeds. BYOL: Bootstrap Your Own Latent; MoCo: Momentum Contrast; SimCLR: simple framework for contrastive learning of visual representations; SimSiam: simple Siamese.

To further investigate the benefits of self-supervised learning, we evaluated the effectiveness of the learned feature representations with varying training data on Sleep EDF (Figure 3). The default setting is to split all the data into pretrain, training, or test sets by 90%: 5%: 5%. In this section, we maintained the 5% test set constant and resplit the pretrain and training sets (after resplitting, we ensured that all the training set data have labels and removed the labels from the pretrain set), such that the training proportion becomes 0.5%, 1%, 2%, 5%, and 10%, and the rest is used for the pretrain set. This resplitting was conducted at the subject level, after which we again segmented each subject’s recording within the pretrain or training set. We compared our ContraWR+ model to MoCo, SimCLR, BYOL, SimSiam, and the supervised baseline models. Similar ablation studies on SHHS and MGH can be found in Multimedia Appendix 4. Our model outperforms the compared models consistently with different amounts of training data. For example, our model achieves similar performance (with only 5% data as training) to that of the best baseline, BYOL, which needs twice the amount of training data (10% data as training). Also, compared to the supervised model, the self-supervised methods performed better when the labels were insufficient; for example, only ≤2% of the data were labeled.

### Representation Projection

We next sought to assess the quality of the learned feature representations. To do this, we used the representations produced by ContraWR+ on the MGH data set and randomly selected 5000 signal epochs per label from the data set. The ContraWR+ encoder is optimized on the pretrain step without using the labels. We extracted feature representations for each sample through the encoder network and used uniform manifold approximation and projection (UMAP) [16] to project onto the 2D space. We finally color-coded samples according to sleep stage labels for illustration.

**Figure 4.**
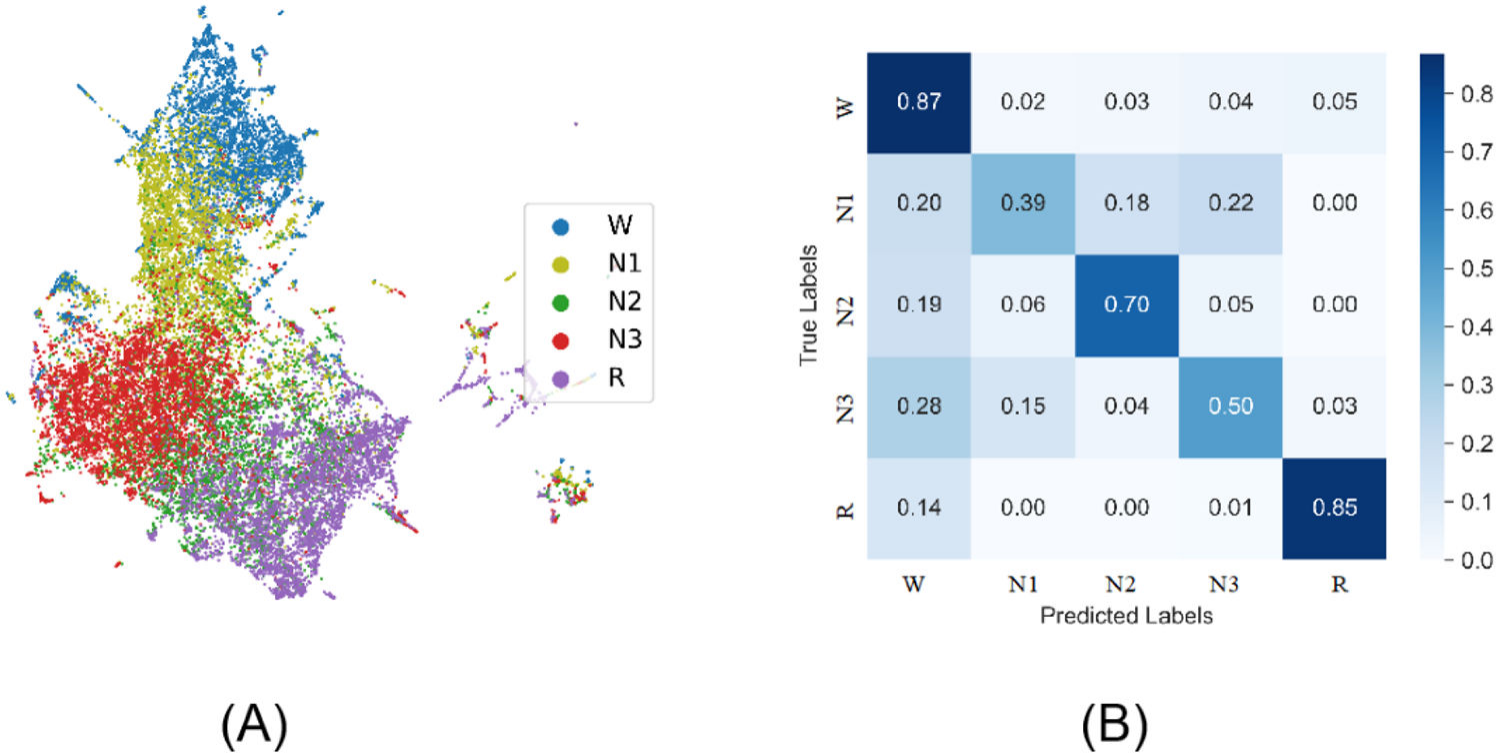
Uniform manifold approximation and projection and confusion matrix. (A) Using the Massachusetts General Hospital’s (MGH’s) MGH Sleep data set, we projected the output representations of each signal into a 2D space and color by the actual labels. (B) We have included a confusion matrix on sleep staging.

The 2D projection is shown in Figure 4. We also computed the confusion matrix from the evaluation stage (based on the test set; also shown in Figure 4). In the UMAP projection, epochs from the same latent class are closely colocated, which implies that the pretrain step extracts important information for sleep stage classification from the raw unlabeled EEG signals. Stage N1 overlaps with stages W, N2, and N3, which is as expected given that N1 is often ambiguous and thus difficult to classify even for well-trained experts [1].

### Hyperparameter Ablation Study

To investigate the sensitivity of our model to hyperparameter settings, we tested with different batch sizes and trained on different values for the Gaussian parameter σ, temperature T, and margin δ. We focused on the ContraWR+ model and evaluated it on the Sleep EDF data set. During the experiment, the default settings are a batch size of 256, σ of 2, T of 2, δ of 0.2, learning rate η of 2×10^−4^, weight decay of 10^−4^, and epoch of 100. When testing on 1 hyperparameter, others are maintained constant.

**Figure 5.**
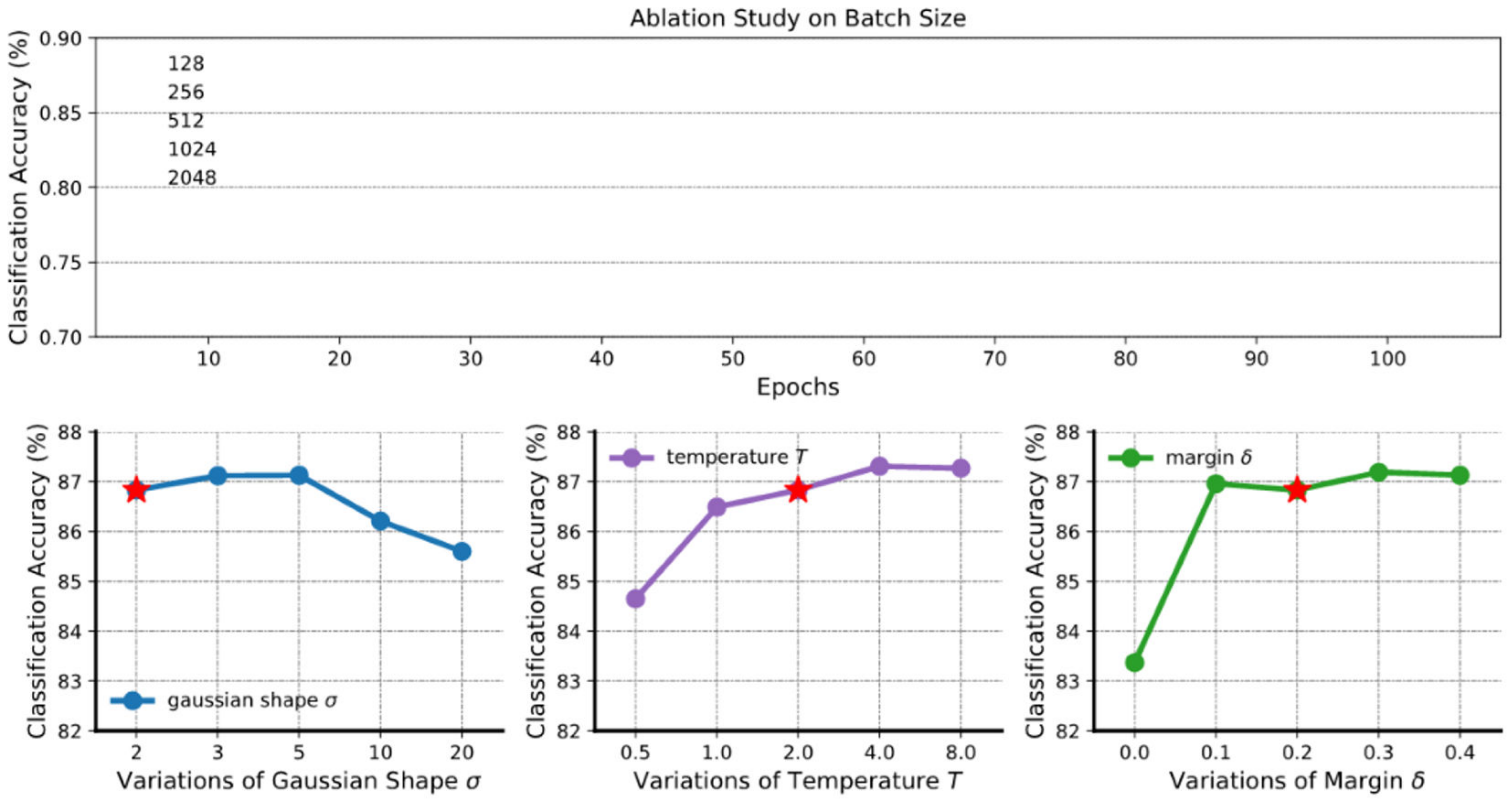
Ablation study on batch size and 3 hyperparameters. The curves indicate the mean values and shaded areas show the SD of training/test over 5 random seeds. The red star denotes the default setting. It is obvious that with a larger batch size, the model will perform better, but it is not sensitive to all hyperparameters.

The ablation study’s results are in shown in Figure 5; the red star indicates the default configuration. Each configuration runs with 5 different random seeds, and the error bars indicate the SD over 5 experiments. We see that the model is not sensitive to batch size. We see that over a large range (<10) the model is insensitive to the Gaussian width σ. For temperature T, we noted previously that a very small T may be problematic, and a very large T reduces ContraWR+ to ContraWR. Based on the ablation experiments, the performance is relatively insensitive to choices of T. For the margin δ, the difference in distance is bounded (given a fixed σ of 2):

$$\|sim(z_{i},z_{w})-sim(z_{i},z_{j})\|\leq {\left\|\text{exp}\left(-\frac{0^{2}}{2\sigma^{2}}\right)-\text{exp}\left(-\frac{2^{2}}{2\sigma^{2}}\right)\right\|}^{2}\approx 0.3935$$

Thus, δ should be large enough; that is, δ≥0.1.

### Ethical Considerations

This study has been approved by the Institutional Review Board of Beth Israel Deaconess Medical Center (BIDMC IRB protocol #2022P000417 [Brain Informatics Database]).

## Discussion

### Principal Results

Our proposed ContraWR and ContraWR+ models outperformed 4 recent self-supervised learning methods on the sleep staging task across 3 large EEG data sets (*P*<.001 in almost all cases). ContraWR+ also superseded supervised learning when fewer training labels were available (eg, a 4% improvement in accuracy when less than 2% of data were labeled). Moreover, the models provided well-separated representative structures in 2D projection.

### Comparison With Prior Work

#### Self-Supervised Learning

Many deep generative methods have been proposed for unsupervised representation learning. They mostly rely on autoencoding [17-19] or adversarial training [20-22]. Mutual information maximization is also popular for compressing input data into a latent representation [23-25].

Recently, self-supervised contrastive learning [2,3,7,8,14] has become popular, where loss functions are devised from representation similarity and negative sampling. However, one recent study [4] highlighted inherent limitations of negative sampling and showed that this strategy could hurt the learned representation significantly [5]. To address these limitations, Chuang et al [5] used the law of total probability and approximated the per-class negative sample distribution using the weighted sum of the global data distribution and the expected class label distribution. However, without the actual labels, the true class label distribution is unknown. Grill et al [7] and Chen and He [8] proposed ignoring negative samples and learning latent representations using only positive pairs.

In this paper, we leverage the negative information by replacing negative samples with the average representation of the batch samples (ie, the world representation). We argue and provide experiments showing that contrasting with the world representation is more powerful and robust in the noisy EEG setting.

#### EEG Sleep Staging

Before the emergence of deep learning, several traditional machine learning approaches [26-28] significantly advanced the field using hand-crafted features, as highlighted by Biswal et al [29]. Recently, deep learning models have been applied to various large sleep databases. SLEEPNET [29] built a comprehensive system combining many machine learning models to learn sleep signal representations. Biswal et al [1] designed a multilayer recurrent and convolutional neural network model to process multichannel signals from EEG. To provide interpretable stage prototypes, Al-Hussaini et al [30] developed a SLEEPER model that uses a particular deep learning approach called prototype learning guided by a decision tree to provide more interpretable results. These studies rely on a large set of labeled training data. However, the annotations are expensive, and oftentimes the labeled set is small. In this study, we exploited the large set of unlabeled data to improve the classification, which is more challenging.

#### Self-Supervised Learning on Physiological Signals

While image [31,32], video [33], language [34,35], and speech [36] representations have benefited from contrastive learning, research on learning physiological signals has been limited [37,38]. Lemkhenter et al [39] proposed phase and amplitude coupling for physiological data augmentation. Banville et al [40] conducted representation learning on EEG signals, and they targeted monitoring and pathology screening tasks, without using frequency information. Cheng et al [41] learned subject-aware representations for electrocardiography data and tested various augmentation methods. While most of these methods are based on pairwise similarity comparison, our model provides contrastive information from global data statistics, providing more robust representations. Also, we extracted signal information from the spectral domain.

### Strengths and Limitations

The strengths of our study are (1) we used 3 real-world data sets collected from different institutes and across different year ranges, and 2 are publicly available; (2) our PSG recordings are diverse and generalizable, including 2 data sets collected at home and 1 collected in the laboratory setting, all having relatively large sizes; (3) we have open-sourced our data processing pipelines and all programs used for his study [42], including the baseline model implementations; and (4) we proposed new data augmentation methods for PSG signals and have systematically evaluated their effectiveness. However, the following limitations of our study should be noted: (1) we fixed the neural network encoder architecture in the study, which we plan to explore using other models including recurrent neural networks in the future; (2) we have used STFT to extract spectrograms, but we may consider alternative techniques such as wavelet transformation in future; and (3) our current data augmentation methods are based on clinical knowledge, and we aim to investigate data-driven approaches to design more effective methods in the future.

### Conclusions

This study is motivated by the need to learn effective EEG representations from large unlabeled noisy EEG data sets. We propose a self-supervised contrastive method, ContraWR, and its enhanced variant, ContraWR+. Instead of creating a large number of negative samples, our method contrasts samples with an average representation of many samples. The model is evaluated on a downstream sleep staging task with 3 real-world EEG data sets. Extensive experiments show that the model is more powerful and robust than multiple baselines including MoCo, SimCLR, BYOL, and SimSiam. ContraWR+ also outperforms the supervised counterpart in label-insufficient scenarios.

## Supplementary Material

appendix 2 Multimedia Appendix 2
Illustration for data augmentations (bandpass filtering, noising, flipping, and shifting).
[PNG File , 368 KB-Multimedia Appendix 2]

appendix 1, 3, 4 Multimedia Appendix 1
Supplementary on model implementation.
[PDF File (Adobe PDF File), 168 KB-Multimedia Appendix 1]
Multimedia Appendix 3
Theoretical loss boundness analysis.
[PDF File (Adobe PDF File), 562 KB-Multimedia Appendix 3]
Multimedia Appendix 4
Results on SHHS and MGH data set during varying the label sizes.
[PDF File (Adobe PDF File), 24 KB-Multimedia Appendix 4]

## Figures and Tables

**Table 1.** Data set statistics.

Name	Location	Channels, n	Recordings, n	Epochs, n	Storage (GB)
Sleep Heart Health Study	At home	2	5445	4,535,949	260
Sleep EDF	At home	2	153	415,089	20
MGH^a^ Sleep	In the laboratory	6	6478	4,863,523	1322

a MGH: Massachusetts General Hospital.

**Table 2.** Class label distribution of the data sets.

Name	Epochs, n (%)				
	W	N1	N2	N3	R
Sleep Heart Health Study	1,306,742 (28.8)	169,021 (3.7)	1,856,130 (40.9)	571,191 (12.6)	632,865 (14.0)
Sleep EDF	285,561 (68.8)	21,522 (5.2)	69,132 (16.6)	13,039 (3.2)	25,835 (6.2)
MGH^a^ Sleep	2,154,540 (44.3)	481,488 (9.9)	700,347 (14.4)	855,980 (17.6)	671,168 (13.8)

a MGH: Massachusetts General Hospital.

**Table 3.** Comparison of sleep staging accuracy with different methods.

Name	Sleep staging accuracy (%), mean (SD)^a^		
	Sleep EDF data set	Sleep Heart Health Study data set	MGH^b^ Sleep data set
Supervised	84.98 (0.3562)	75.61 (0.9347)	69.73 (0.4324)
Untrained Encoder	77.83 (0.0232)	60.03 (0.0448)	55.64 (0.0082)
MoCo^c^	85.58 (0.7707)	77.10 (0.2743)	62.14 (0.7099)
SimCLR^d^	83.79 (0.3532)	76.61 (0.3007)	67.32 (0.7749)
BYOL^e^	85.61 (0.7080)	76.64 (0.3783)	70.75 (0.1461)
SimSiam^f^	84.78 (0.8028)	74.25 (0.4796)	62.08 (0.4902)
AVG-KNN-Top1^g^	80.39 (1.3721)	69.70 (0.8944)	60.73 (0.7423)
AVG-KNN-Top5	83.24 (0.6182)	75.18 (0.7845)	69.14 (0.3393)
AVG-KNN-Top50	86.35 (0.3246)	77.63 (0.3625)	71.95 (0.3482)
ContraWR^h^	85.94 (0.2326)	77.52 (0.5748)	71.97 (0.1774)
ContraWR+	86.90 (0.2288)	77.97 (0.2693)	72.03 (0.1823)

a Calculated over 5 random seeds.

b MGH: Massachusetts General Hospital.

c MoCo: Momentum Control.

d SimCLR: simple framework for contrastive learning of visual representations.

e BYOL: Bootstrap Your Own Latent.

f SimSam: simple Siamese.

g AVG-KNN-TopX: average k-nearest neighbor TopX.

h ContraWR: Contrast with the World Representation.

**Table 4.** Evaluation accuracy of different augmentations.

Augmentations	Accuracy (%), mean (SD)^a^
Bandpass	84.23 (0.2431)
Noising	83.60 (0.1182)
Shifting	84.65 (0.2844)
Bandpass + flipping	85.77 (0.2337)
Noising + flipping	84.45 (0.1420)
Shifting + flipping	85.13 (0.0558)
Bandpass + noising	85.37 (0.1214)
Noising + shifting	84.78 (0.1932)
Shifting + bandpass	85.25 (0.1479)
Bandpass + noising + flipping	85.76 (0.1794)
Noising + shifting + flipping	85.17 (0.2301)
Shifting + bandpass + flipping	86.38 (0.2789)

a Calculated over 5 random seeds.

## Abbreviations

AASM: American Academy of Sleep Medicine
BYOL: Bootstrap Your Own Latent
ContraWR: Contrast with the World Representation
EEG: electroencephalogram
EMA: exponential moving average
MGH: Massachusetts General Hospital
MoCo: Momentum Contrast
PSG: polysomnography
REM: rapid eye movement
SHHS: Sleep Heart Health Study
SimCLR: simple framework for contrastive learning of visual representations
SimSiam: simple Siamese
STFT: short-time Fourier transform
UMAP: uniform manifold approximation and projection
