# Supplementary figures and images for: Self-Supervised Electroencephalogram Representation Learning for Automatic Sleep Staging: Model Development and Evaluation Study
Source: JMIR AI. Author manuscript; Available in PMC 2023 Dec 12. (PMC10715804; doi:10.2196/46769)

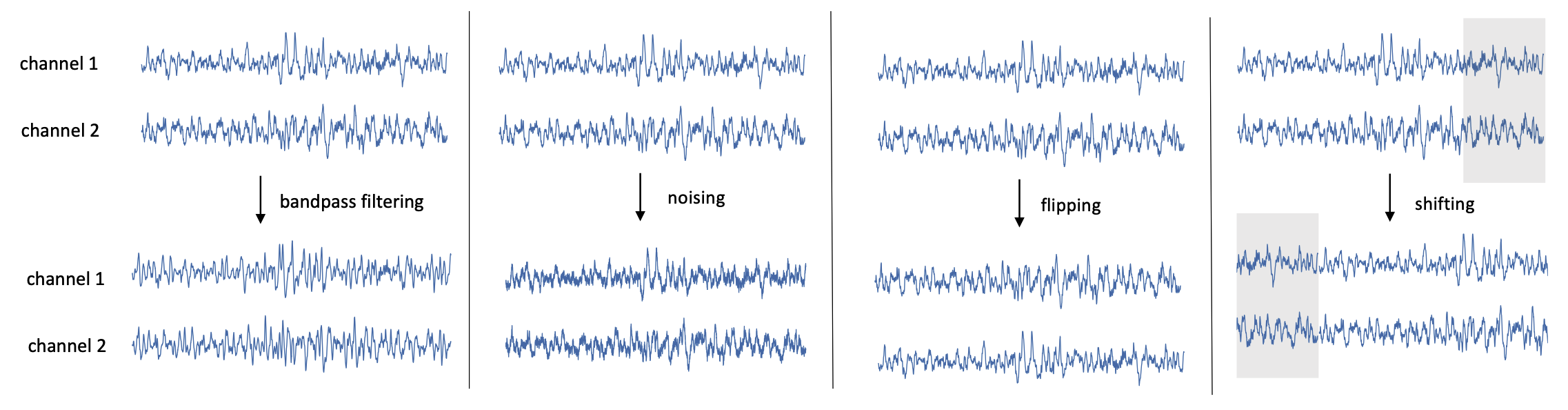

Supplement: appendix 2 — Multimedia Appendix 2 Illustration for data augmentations (bandpass filtering, noising, flipping, and shifting). [PNG File , 368 KB-Multimedia Appendix 2] [file NIHMS1937931-supplement-appendix_2.png]
