# Supplementary material for: Self-Supervised Electroencephalogram Representation Learning for Automatic Sleep Staging: Model Development and Evaluation Study
Source: JMIR AI. Author manuscript; Available in PMC 2023 Dec 12. (PMC10715804; doi:10.2196/46769)
Supplement: appendix 1, 3, 4 — Multimedia Appendix 1 Supplementary on model implementation. [PDF File (Adobe PDF File), 168 KB-Multimedia Appendix 1] Multimedia Appendix 3 Theoretical loss boundness analysis. [PDF File (Adobe PDF File), 562 KB-Multimedia Appendix 3] Multimedia Appendix 4 Results on SHHS and MGH data set during varying the label sizes. [PDF File (Adobe PDF File), 24 KB-Multimedia Appendix 4] [file NIHMS1937931-supplement-appendix_1__3__4.pdf]

Data augmentation

The human brain EEG signals are typically identified by different frequencies based on various sleep stages. In our paper, we consider the following four augmentation methods:

Bandpass Filtering. To reduce noise, we use the order-1 Butterworth filter (implemented by $scipy.signal.butter$), only the within-band frequency is preserved after augmentation. For Sleep EDF, we use frequency band interval (1, 5) and (30, 49); For SHHS, we use interval (1, 3) and (30, 60); For MGH Sleep, we only use interval (0, 30).

Noising. We add independent and identically distributed high-frequency or low-frequency noise onto each channel. For three datasets, the high-frequency signal is sampled from uniform distribution modulated by a noise degree, following the equation:

$$noise\_seq = D * A * \ uniform\_random\_seq,$$

Where D is the noise degree (we use D=0.05 for all datasets), A is the amplitude range of the original signal, $uniform\_random\_seq$ is an independent and identically distributed (i.i.d.) sequence that has the same length as the signal and is generated from a uniform distribution in (-1,1) by $np.random.rand$. To generate the low-frequency noise, we first sample a random noise sequence with $\frac{1}{100}$ of the signal length in the same way, and we later use $scipy.interpolate.interp1d$ to interpolate the noise sequence into the same length, whose frequency will turn low. After generating the noise sequence, we will add the noise to the original signal, where the probability of adding "high" or "low" or "both" will be equal.

Channel Flipping. The sensor on the left side and the right of the brain are placed symmetrically. Thus, we flip the corresponding channels as another augmentation method. For Sleep EDF, we

can flip the Fpz-Cz and Pz-Oz channels; For MGH Sleep, we can flip the F3-M2 and F4-M1, or C3-M2 and C4-M1 or O1-M2 and O2-M1; For SHHS, we can flip C3/A2 and C4/A1.

Shifting. Within one instance, we will rotate/delay the signal for a certain time span. For three datasets, we uniformly split a signal epoch into two pieces and then resemble it as the augmentation. An illustration is provided in Figure 1. After doing augmentations, we will clip the signal amplitudes within a valid sensing range (which is the max measured signal amplitude, 2.5e-4 for Sleep EDF, 50 for MGH, 1.25e-4 for SHHS). To get an augmented version of a signal, we randomly apply one of the augmentation methods with equal probability.

Model Implementation

All models are implemented using PyTorch 1.4.0 and optimized with Adam optimizer, 2e-4 as learning rate, 1e-4 as weight decay. For all datasets, we use 256 as batch size and use $\sigma = 2, \delta = 0.2, T = 2$ as the hyperparameters. In terms of the dimension of learned representations, 128 is for Sleep EDF, 256 for SHHS, and 192 for MGH. We implement the logistic regression model by scikit-learn with default setting and 500 as the maximum iteration for logistic regression evaluation of contrastive methods (the logistic regression is implemented by $klearn.linear\_model.LogisticRegression$ with $max\_iter = 500$ argument.). The experiments are conducted on two NVIDIA GTX 3090 GPUs with 24GB memory each, a 32-core CPU Linux machine with 256GB RAM. Note that the training process is IO-intensive, which involves loading sample files from the disk batch-by-batch. Therefore, a 4TB SSD persistent disk is used to store the raw signal epochs.

Appendix 3

Self-supervised EEG Representation Learning for Automatic Sleep Staging (Loss Analysis Appendix)

Chaoqi Yang chaoqiy2@illinois.edu
University of Illinois at Urbana-Champaign

Danica Xiao danica.xiao@gmail.com
Amplitude

M. Brandon Westover mwestover@mgh.harvard.edu
Massachusetts General Hospital, Harvard Medical School

Jimeng Sun jimeng.sun@gmail.com
University of Illinois at Urbana-Champaign

Abstract

In this appendix, we show that our loss function can be decomposed into two parts "Alignment" and "Uniformity", which has been confirmed to be essential in self-supervised learning (SSL). We also provide theoretical and empirical supports for these two properties. We opened our codes and pretrained models are provided in Github [1].

1 `ContraWR`: Contrast with the World Representation

In contrastive learning, the positive pairs are generally valid (from data augmentations) for computing the loss, however, "negative" samples are conceived (people usually call random samples as "negative" samples). Few works have been done to improve the existing negative sampling strategy or find effective alternatives. Among the pioneers, Chuang et al. (2020) seeks to approximate an unbiased negative sample distribution from the world distribution and the class distribution, however, the real class distributions are usually unknown given an unlabeled dataset. Recently, BYOL (Grill et al., 2020) and SimSiam (Chen and He, 2020) are proposed, which ignores negative sampling (or contrastive information) and exploits only the positive samples. However, contrastive information could be beneficial if used properly.

We utilize the global statistics of the world/dataset and propose a new contrastive method, `ContraWR`. In our method, a large number of negative samples are replaced by a simple average representation of the negative samples, which is called *the world representation*. The world representation makes the pretrain step much more robust. **In fact, the similarity between positive pair $(\mathbf{z}', \mathbf{z}'')$ should be stronger than most of the random pairs $(\mathbf{z}', \mathbf{z}_w)$ but not all, which is an improper assumption in recent works (He et al., 2020a,b; Chen et al., 2020).** Our pretrain step therefore follows a weaker guidance for the contrastive problem: *the similarity between positive pairs is stronger than the similarity between the corresponding instance and the average of the world/dataset.*

The `ContraWR` model is shown in Figure 1. The encoder network $f(\cdot)$ maps two different views of the same instance to latent representations. Then, the project head $g(\cdot)$ further projects the latent representations onto a unit hypersphere, where the loss is defined. Though we only show one encoder $f(\cdot)$ and one projector $g(\cdot)$, our `ContraWR` can also fit into two-network pipelines, where an online network $f_\theta(\cdot), g_\theta(\cdot)$ and a momentum target network $f_\phi(\cdot), g_\phi(\cdot)$ are presented. We try both in the experiment.

[1] https://github.com/ycq091044/ContraWR

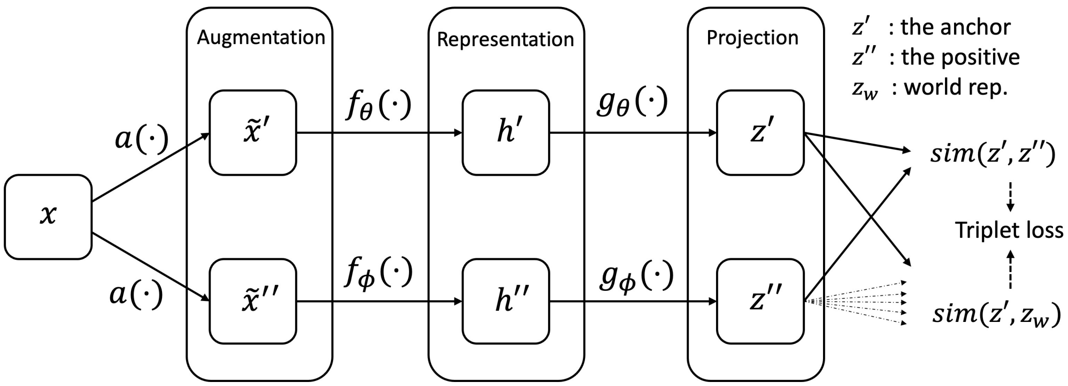

Figure 1: `ContraWR` Pipeline

The world representation. Assume $\mathbf{z}'$ is the anchor, $\mathbf{z}''$ is the positive sample, and $\mathbf{z}_k$ is a generic random sample. For the anchor $\mathbf{z}'$, we create a world representation (average of the negative samples), $\mathbf{z}_w$, as the *only* contrastive information. To formalize, we assume $p(\cdot)$ is the sample distribution over the dataset, independent to $\mathbf{z}'$. The world representation $\mathbf{z}_w$ is defined by,

$$\mathbf{z}_w = E_{k\sim p(\cdot)}[\mathbf{z}_k] = E_{\mathbf{z}_k\sim p(\cdot)}[\mathbf{z}_k]. \tag{1}$$

Here, we denote $\mathcal{D} = \{\mathbf{x} : \|\mathbf{x}\| \leq 1,\ \mathbf{x} \in \mathbb{R}^m\}$. Obviously, $\mathbf{z}_w \in \mathcal{D}$. We assume the joint distribution of positive pairs as $p^+(\cdot,\cdot)$, where its marginal distrbution is naturally $p(\cdot)$.

Gaussian kernel measure. Instead of using cosine similarity, this paper adopts Gaussian kernel defined on $\mathcal{D}$, $Ker(\mathbf{x},\mathbf{y}) :\ \mathcal{D} \times \mathcal{D} \mapsto (0,1]$ as a similarity measure. Formally, given two projections $\mathbf{z}', \mathbf{z}'' \in \mathbb{S}^{m-1} \subset \mathcal{D}$, the similarity is defined as,

$$sim(\mathbf{z}',\mathbf{z}'') = Ker(\mathbf{z}',\mathbf{z}'') = \exp\left(-\frac{\|\mathbf{z}' - \mathbf{z}''\|^2}{2\sigma^2}\right) \tag{2}$$

We use $\sigma = 2$ throughout the paper and also in experiments. For the selection of σ, we will later specify two criterions: (i) $\sigma \geq 2$; (ii) σ should be empirically small.

Loss function. For an random draw from the joint distribution $(\mathbf{z}',\mathbf{z}'') \sim p^+$, where $(\mathbf{z}',\mathbf{z}'')$ is a positive pair. We devise a triplet loss for the pair,

$$\mathcal{L} = [sim(\mathbf{z}',\mathbf{z}_w) + \delta - sim(\mathbf{z}',\mathbf{z}'')]_+ \tag{3}$$

where we regard $\mathbf{z}'$ as the anchor, $\mathbf{z}''$ as the positive sample and $\delta > 0$ is the empirical margin. The loss is minimized over batches when similarity of positive pairs, $sim(\mathbf{z}',\mathbf{z}'')$, is larger than similarity to the world representation, $sim(\mathbf{z}',\mathbf{z}_w)$, by a margin of δ. In fact, in the appendix, we show that the results are not sensitive to the non-negativity function and margin δ. However, some may find the margin-based form useful, so we keep it as a triplet loss.

Analysis of the loss. Without loss of generality, let us first skip the non-negativity function and δ in Equation (3), and room in to the expectation over the joint distribution (suppose a batch with infinity size),

$$E_{(\mathbf{z}',\mathbf{z}'')\sim p^+(\cdot,\cdot)}[sim(\mathbf{z}',\mathbf{z}_w) - sim(\mathbf{z}',\mathbf{z}'')] = E_{(\mathbf{z}',\mathbf{z}'')\sim p^+(\cdot,\cdot)}[sim(\mathbf{z}', E_{k\sim p(\cdot)}[\mathbf{z}_k]) - sim(\mathbf{z}',\mathbf{z}'')] \tag{4}$$

Since $sim(\cdot,\cdot)$ is bounded, we could take the expectations separately and re-arrange the formula,

$$-\underbrace{E_{(\mathbf{z}',\mathbf{z}'')\sim p^{+}(\cdot,\cdot)}[sim(\mathbf{z}',\mathbf{z}'')]}_{Alignment}+\underbrace{E_{(\mathbf{z}',\mathbf{z}'')\sim p^{+}(\cdot,\cdot)}[sim(\mathbf{z}',E_{k\sim p(\cdot)}[\mathbf{z}_k])]}_{Uniformity} \tag{5}$$

In light of recent works (Wang and Isola, 2020; Chen and Li, 2020), who identify two key properties of contrastive loss: Alignment and Uniformity. We reveal that our design coincides with these two properties.

Alignment requires the encoder to assign similar features to similar instances. In Equation (5), the first term is straightforwardly defined with the expected similarity between positive pairs. Minimizing the above equation would enforce the encoder to map similar input samples onto near region.

Uniformity prefers a feature distribution with high entropy on the unit hypersphere, which preserves maximal information. Let us name the second term (of Equation (5)) $\mathcal{L}_2$, and do the following derivation,

$$\mathcal{L}_2 = E_{(\mathbf{z}',\mathbf{z}'')\sim p^{+}(\cdot,\cdot)}[sim(\mathbf{z}',E_{k\sim p(\cdot)}[\mathbf{z}_k])] \tag{6}$$

$$= E_{\mathbf{z}'\sim p(\cdot)}[sim(\mathbf{z}',E_{k\sim p(\cdot)}[\mathbf{z}_k])] \tag{7}$$

$$= E_{\mathbf{z}'\sim p(\cdot)}\left[\exp\left(-\frac{1}{2\sigma^2}\left\|\mathbf{z}'-E_{k\sim p(\cdot)}[\mathbf{z}_k]\right\|^2\right)\right] \tag{8}$$

$$\geq \exp\left(-\frac{1}{2\sigma^2}\cdot E_{\mathbf{z}'\sim p(\cdot)}\left[\left\|\mathbf{z}'-E_{k\sim p(\cdot)}[\mathbf{z}_k]\right\|^2\right]\right) \tag{9}$$

$$= \exp\left(-\frac{1}{2\sigma^2}\cdot\left(1-\langle E_{\mathbf{z}'\sim p(\cdot)}[\mathbf{z}'],E_{\mathbf{z}_k\sim p(\cdot)}[\mathbf{z}_k]\rangle\right)\right) \tag{10}$$

$$= \exp\left(\frac{\|E_{\mathbf{z}'\sim p(\cdot)}[\mathbf{z}']\|^2-1}{2\sigma^2}\right). \tag{11}$$

From Equation (8) to (9), we use Jensen's inequality since $\exp(\cdot)$ is a convex function. In Equation (9), we could observe that $\mathcal{L}_2$ is the upper bound of the term, $E_{\mathbf{z}'\sim p(\cdot)}\left[\left\|\mathbf{z}'-E_{k\sim p(\cdot)}[\mathbf{z}_k]\right\|^2\right]$, which is negatively correlated to the variance-like objection over all the projections (since $\mathbf{z}$ is restricted onto the hypersphere, the expectation term is not exactly the sum of independent variance over all projected dimensions). In Equation (11), the term $\|E_{\mathbf{z}'\sim p(\cdot)}[\mathbf{z}']\|^2$ is the $L2$ norm of the world representation. Conceptually, by minimizing the upper bound $\mathcal{L}_2$, the world representation will be restricted (see Equation (11)), and the joint variance of all feature dimensions is enlarged (see Equation (9)).

Wang and Isola (2020) proves that the unique minimizer of the following *average Gaussian potential* is the normalized surface area measure (i.e., $p(\cdot)$ is the uniform distribution) on $\mathbb{S}^{m-1}$,

$$\mathcal{L}_{gaussian} = E_{\mathbf{z}'\sim p(\cdot),\ \mathbf{z}_k\sim p(\cdot)}\left[\exp(-t\|\mathbf{z}'-\mathbf{z}_k\|^2)\right], \quad t>0. \tag{12}$$

In fact, $\mathcal{L}_2$ is also an upper bound of $\mathcal{L}_{gaussian}$ given $t=\frac{1}{2\sigma^2}$.

Proposition 1. *For $\mathcal{D}=\{\mathbf{x}:\|\mathbf{x}\|\leq 1,\ \mathbf{x}\in\mathbb{R}^m\}$, given a fixed $\mathbf{y}\in\mathcal{D}$, given $\sigma\geq 2$, for any $\mathbf{x}\in\mathcal{D}$, the following kernel-induced function is concave,*

$$h(\mathbf{x}) = \exp\left(-\frac{1}{2\sigma^2}\|\mathbf{y}-\mathbf{x}\|^2\right). \tag{13}$$

Based on Proposition 1 (see proof in Appendix A), we apply Jensen's inequality on Equation (8)

and reach the following result,

$$\mathcal{L}_2 \geq E_{\mathbf{z}' \sim p(\cdot),\ \mathbf{z}_k \sim p(\cdot)} \left[\exp\left(-\frac{1}{2\sigma^2} \left\| \mathbf{z}' - \mathbf{z}_k \right\|^2 \right) \right] = \mathcal{L}_{gaussian}. \tag{14}$$

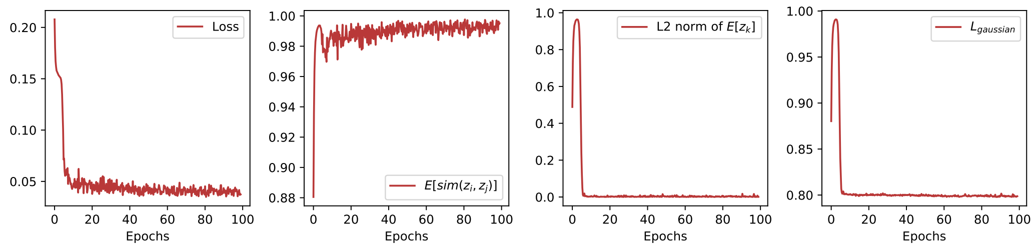

Figure 2: Statistical Curves During Training on Sleep EDF Dataset

Empirically, we shown (i) the actually loss function; (ii) average similarity of positive pairs; (iii) $L2$ norm of the world representation; (iv) average Gaussian potential $\mathcal{L}_{gaussian}$ in Figure 2. Clearly, durign training, the loss is decreasing, and the average similarity between positive pairs are gradually approaching 1. With only a few iterations, the world representation is restricted to the original, which means all projected vectors lie on the unit hypersphere symmetrically, indicating a high entropy and non-collapse. Interestingly, the average Gaussian potential is gradually decreasing below 0.8, while $\exp(-\frac{1}{4}) \approx 0.78$ is the exact average Gaussian potential for uniform distribution over the hypersphere, since at that case, any two random unit vectors would be orthogonal almost surely. In sum, by minimizing the contrastive loss in Equation (4), or equivalently Equation (5), the alignment and uniformity properties are obtained.

2 ContraWR$^+$: Contrast with Instance-aware World Representation

Harder criterion. To learn a better representation, we find that a harder criterion would be more beneficial: *the similarity between positive pairs is stronger than the similarity between the corresponding instance and the weighted average of the world/dataset, where the weight is higher for closer samples.* Robinson et al. (2020) also showed that harder samples are more helpful.

Instance-aware world representation. In this section, the world representation is enhanced by modifying the sampling distribution to be instance-specific. We assume $p(\cdot \mid \mathbf{z})$ as the instance-aware sampling distribution, opposed to the instance-independent sample distribution $p(\cdot)$,

$$p(\cdot \mid \mathbf{z}) \propto \exp\left(\frac{\langle \cdot, \mathbf{z} \rangle}{T} \right) \tag{15}$$

where $T \geq 0$ is a temperature hyperparameter. The similar samples are selected with higher probability parametrized by $p(\cdot \mid \mathbf{z})$. Accordingly, for an anchor $\mathbf{z}_i$, the instance-aware world representation changes into,

$$\mathbf{z}_w = E_{k \sim p(\cdot \mid \mathbf{z}_i)}[\mathbf{z}_k] = \frac{E_{k \sim p(\cdot)} \left[\exp\left(\frac{\langle \mathbf{z}_k, \mathbf{z}_i \rangle}{T} \right) \cdot \mathbf{z}_k \right]}{E_{k \sim p(\cdot)} \left[\exp\left(\frac{\langle \mathbf{z}_k, \mathbf{z}_i \rangle}{T} \right) \right]} \tag{16}$$

where T controls the contrastive hardness of the world representation. In fact, when $T \to \infty$, $p(\cdot \mid \mathbf{z})$ is identical to $p(\cdot)$ and Equation (16) reduces to the the simple version in Equation (1);

while $T \to 0^+$, the form becomes trivial, $\mathbf{z}_w = argmax_{\mathbf{z}_k}(sim(\mathbf{z}_i, \mathbf{z}_k))$. We have tested different T in Section **??**, and find the model is not sensitive to T within a wide range.

Let us re-write the similarity measure given the anchor $\mathbf{z}_i$ and the instance-aware world representation $\mathbf{z}_w$,

$$sim(\mathbf{z}_i, \mathbf{z}_w) = sim\left(\mathbf{z}_i, E_{k\sim p(\cdot|\mathbf{z}_i)}[\mathbf{z}_k]\right) = \exp\left(-\frac{1}{2\sigma^2}\left\|\mathbf{z}_i - E_{k\sim p(\cdot|\mathbf{z}_i)}[\mathbf{z}_k]\right\|^2\right) \tag{17}$$

Analysis of harder contrastive loss. In this scenario, the analysis starts from the same point as Equation (5). The first (alignment) term remains the same, and for the second (uniformity) term, it changes into,

$$\mathcal{L}_2 = E_{(\mathbf{z}',\mathbf{z}'')\sim p^+(\cdot,\cdot)}[sim(\mathbf{z}_i, E_{k\sim p(\cdot|\mathbf{z}_i)}[\mathbf{z}_k])] \tag{18}$$

$$= E_{\mathbf{z}'\sim p(\cdot)}\left[\exp\left(-\frac{1}{2\sigma^2}\left\|\mathbf{z}_i - E_{\mathbf{z}_k\sim p(\cdot|\mathbf{z}_i)}[\mathbf{z}_k]\right\|^2\right)\right] \tag{19}$$

$$\geq E_{\mathbf{z}'\sim p(\cdot),\ \mathbf{z}'_k\sim p(\cdot|\mathbf{z}_i)}\left[\exp\left(-\frac{1}{2\sigma^2}\|\mathbf{z}_i - \mathbf{z}_k\|^2\right)\right] \tag{20}$$

$$= E_{(\mathbf{z}',\mathbf{z}_k)\sim\exp\left(\frac{\langle\mathbf{z}_i,\mathbf{z}_k\rangle}{T}\right)}\left[\exp\left(-\frac{1}{2\sigma^2}\|\mathbf{z}_i - \mathbf{z}_k\|^2\right)\right] \tag{21}$$

$$\geq E_{\mathbf{z}'\sim p(\cdot),\ \mathbf{z}_k\sim p(\cdot)}\left[\exp\left(-\frac{1}{2\sigma^2}\|\mathbf{z}_i - \mathbf{z}_k\|^2\right)\right] \tag{22}$$

From Equation (19) to (20), we also use Jensen's inequality based on the concave property in Proposition 1. We observe that the new (harder) $\mathcal{L}_2$ upper bounds a *more strict (instance-aware) Gaussian potential* in Equation (21), which itself is the upper bound of the *average Gaussian potential* in Equation (22) (Wang and Isola, 2020). The new loss function empirically leads to better performance in the experiment.

3 Code Snippets

The exact PyTorch snippets are attached as reference,

```
# emb_ahr, emb_pos   :Nx128, anchor and positive projections
# delta, T, sigma    :margin, temperature, Gaussian shape

# gaussian kernel
def gaussian(z_i, z_j):  # z_i: Nx128, z_j: Nx128 or 1x128
    mse = torch.sum(torch.pow(z_i - z_j, 2), dim=1)  # Nx1
    return torch.exp(-mse / (2 * sigma**2))  # Nx1

# --------- Contrast with the World Representation ---------
# compute z_w
z_w=torch.mean(emb_pos, dim=0)  # 1x128

# compute sim(z_i,z_j) and sim(z_i,z_w)
sim_pos = gaussian(emb_ahr, emb_pos)  # Nx1
sim_wld = gaussian(emb_ahr, z_w)      # Nx1

# loss function
zero_vec = torch.zeros(sim_pos.shape).to(device)
loss = torch.max(zero_vec, sim_wld + delta - sim_pos).mean()

# ---- Contrast with Instance-aware World Representation ----
# compute z_w
dot_sim = torch.mm(emb_ahr, emb_pos.t())      # NxN
```

```
weight = torch.nn.Softmax(dim=1)(dot_sim/T)    # NxN
z_w = torch.mm(weight, emb_pos)                # Nx128

# compute sim(z_i,z_j) and sim(z_i,z_w)
sim_pos = gaussian(emb_ahr, emb_pos)  # Nx1
sim_wld = gaussian(emb_ahr, z_w)      # Nx1

# loss function
zero_vec = torch.zeros(sim_pos.shape).to(device)
loss = torch.max(zero_vec, sim_wld + delta - sim_pos).mean()
```

References

Ting Chen and Lala Li. Intriguing properties of contrastive losses. *arXiv preprint arXiv:2011.02803*, 2020.

Ting Chen, Simon Kornblith, Mohammad Norouzi, and Geoffrey Hinton. A simple framework for contrastive learning of visual representations. In *Int. Conference on Machine Learning (ICML)*, pages 10709–10719, 2020.

Xinlei Chen and Kaiming He. Exploring simple siamese representation learning, 2020.

Ching-Yao Chuang, Joshua Robinson, Lin Yen-Chen, Antonio Torralba, and Stefanie Jegelka. Debiased Contrastive Learning. In *Advances in Neural Information Processing Systems (NeurIPS)*, 2020.

Jean-Bastien Grill, Florian Strub, Florent Altché, Corentin Tallec, Pierre Richemond, Elena Buchatskaya, Carl Doersch, Bernardo Avila Pires, Zhaohan Guo, Mohammad Gheshlaghi Azar, et al. Bootstrap your own latent-a new approach to self-supervised learning. *Advances in Neural Information Processing Systems*, 33, 2020.

Kaiming He, Haoqi Fan, Yuxin Wu, Saining Xie, and Ross Girshick. Momentum contrast for unsupervised visual representation learning. In *IEEE Conference on Computer Vision and Pattern Recognition (CVPR)*, 2020a.

Kaiming He, Haoqi Fan, Yuxin Wu, Saining Xie, and Ross Girshick. Momentum contrast for unsupervised visual representation learning. In *IEEE Conference on Computer Vision and Pattern Recognition (CVPR)*, pages 9729–9738, 2020b.

Joshua Robinson, Ching-Yao Chuang, Suvrit Sra, and Stefanie Jegelka. Contrastive learning with hard negative samples. *arXiv preprint arXiv:2010.04592*, 2020.

Tongzhou Wang and Phillip Isola. Understanding contrastive representation learning through alignment and uniformity on the hypersphere. In *Int. Conference on Machine Learning (ICML)*, pages 9574–9584, 2020.

A Proof of Concavity

Proposition 1. *Assume $\mathcal{D} = \{\mathbf{x} : \|\mathbf{x}\| \leq 1,\ \mathbf{x} \in \mathbb{R}^m\}$. Given a fixed $\mathbf{y} \in \mathcal{D}$, given $\sigma \geq 2$, for any $\mathbf{x} \in \mathcal{D}$, the following kernel-induced function is concave,*

$$h(\mathbf{x}) = \exp\left(-\frac{1}{2\sigma^2}\|\mathbf{y} - \mathbf{x}\|^2\right). \tag{23}$$

Proof. Since $f(\mathbf{x})$ is a real-valued function, and the domain $\mathcal{D}$ is a convex set, the above proposition is equivalent to: for any $\mathbf{x}_1, \mathbf{x}_2 \in \mathcal{D}$, for any $\lambda \in [0, 1]$,

$$h((1-\lambda)\mathbf{x}_1 + \lambda\mathbf{x}_2) \geq (1-\lambda)h(\mathbf{x}_1) + \lambda h(\mathbf{x}_2). \tag{24}$$

We introduce new vectors (reducing offset $\mathbf{y}$) and new functions,

$$\mathbf{y}_1 = \mathbf{y} - \mathbf{x}_1 \tag{25}$$

$$\mathbf{y}_2 = \mathbf{y} - \mathbf{x}_2 \tag{26}$$

$$g(t) = \exp\left(-\frac{t^2}{2\sigma^2}\right), \quad \|t\| \leq 2 \tag{27}$$

Thus, Equation (24) could be re-written into,

$$g\left(\|(1-\lambda)\mathbf{y}_1 + \lambda\mathbf{y}_2\|\right) \geq (1-\lambda)g(\|\mathbf{y}_1\|) + \lambda g(\|\mathbf{y}_2\|). \tag{28}$$

Two step proof. We prove this inequality by two steps. First, we prove that

$$g\left(\|(1-\lambda)\mathbf{y}_1 + \lambda\mathbf{y}_2\|\right) \geq g\left((1-\lambda)\|\mathbf{y}_1\| + \lambda\|\mathbf{y}_2\|\right) \tag{29}$$

It is obvious, since for any $0 \leq a \leq b \leq 2$, we have $g(a) \geq g(b)$. Also, by Triangle inequality, we have,

$$\|(1-\lambda)\mathbf{y}_1 + \lambda\mathbf{y}_2\| \leq \|(1-\lambda)\mathbf{y}_1\| + \|\lambda\mathbf{y}_2\| = (1-\lambda)\|\mathbf{y}_1\| + \lambda\|\mathbf{y}_2\| \tag{30}$$

By the way,

$$(1-\lambda)\|\mathbf{y}_1\| + \lambda\|\mathbf{y}_2\| \leq max_{\mathbf{x},\mathbf{y}\in\mathcal{D}}\|\mathbf{y} - \mathbf{x}\| = 2. \tag{31}$$

Second, we prove that

$$g\left((1-\lambda)\|\mathbf{y}_1\| + \lambda\|\mathbf{y}_2\|\right) \geq (1-\lambda)g(\|\mathbf{y}_1\|) + \lambda g(\|\mathbf{y}_2\|). \tag{32}$$

Since $0 \leq \|\mathbf{y}_1\|, \|\mathbf{y}_2\| \leq 2$, Equation (32) is equivalent to prove that function $g(t)$ is concave in $[-2, 2]$. The second derivative of $g(t)$ is calculated as,

$$g''(t) = \frac{t^2 - \sigma^2}{\sigma^4} \cdot \exp\left(-\frac{t^2}{2\sigma^2}\right) \tag{33}$$

We could tell that if $\sigma \geq 2$, then $g''(t) \leq 0$, which makes $g(t)$ concave. □

Appendix 4

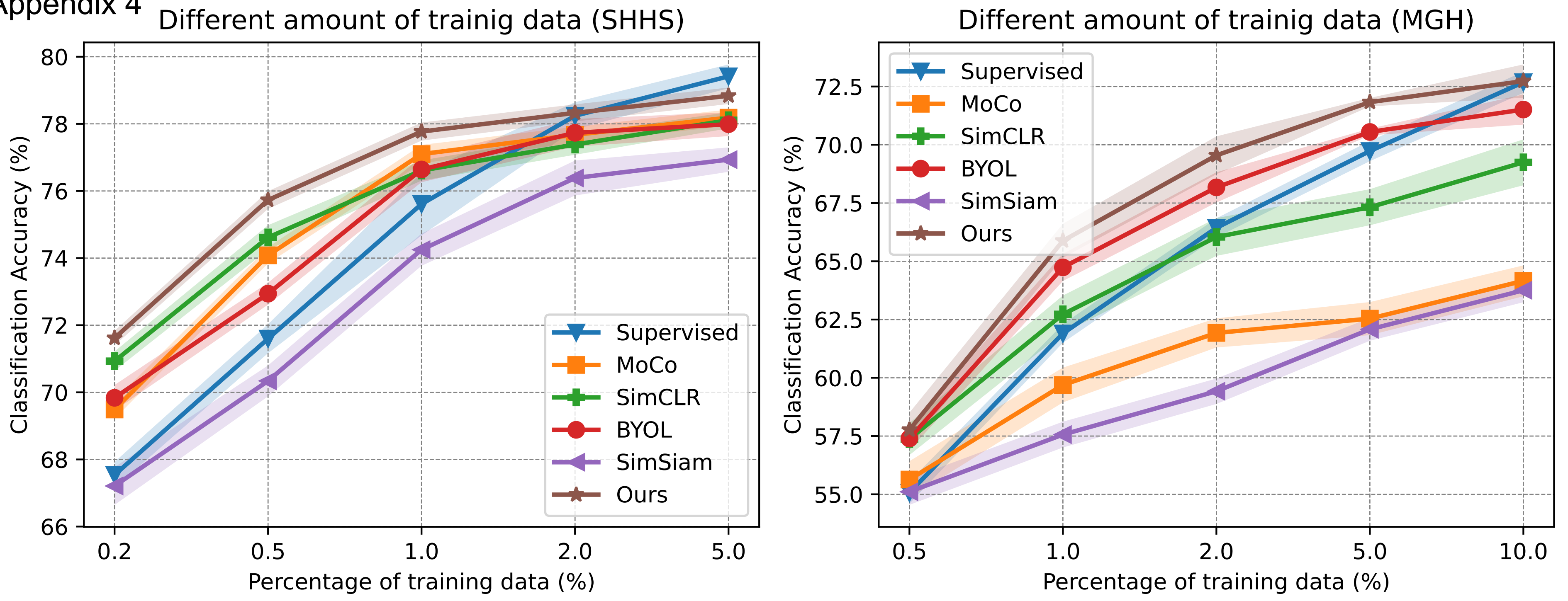
Different amount of trainig data (SHHS)
Classification Accuracy (%)
Percentage of training data (%)
66
68
70
72
74
76
78
80
0.2
0.5
1.0
2.0
5.0
Supervised
MoCo
SimCLR
BYOL
SimSiam
Ours
Different amount of trainig data (MGH)
Classification Accuracy (%)
Percentage of training data (%)
55.0
57.5
60.0
62.5
65.0
67.5
70.0
72.5
0.5
1.0
2.0
5.0
10.0
Supervised
MoCo
SimCLR
BYOL
SimSiam
Ours
