# Supplementary material for: Self-Supervised Electroencephalogram Representation Learning for Automatic Sleep Staging: Model Development and Evaluation Study
Source: JMIR AI. 2023 Jul 26;2:e46769. doi: 10.2196/46769 (PMC10715804; doi:10.2196/46769)
Supplement: Supplementary file 1 [file ai_v2i1e46769_app1.pdf]

Data augmentation

The human brain EEG signals are typically identified by different frequencies based on various sleep stages. In our paper, we consider the following four augmentation methods:

Bandpass Filtering. To reduce noise, we use the order-1 Butterworth filter (implemented by $scipy.signal.butter$), only the within-band frequency is preserved after augmentation. For Sleep EDF, we use frequency band interval (1, 5) and (30, 49); For SHHS, we use interval (1, 3) and (30, 60); For MGH Sleep, we only use interval (0, 30).

Noising. We add independent and identically distributed high-frequency or low-frequency noise onto each channel. For three datasets, the high-frequency signal is sampled from uniform distribution modulated by a noise degree, following the equation:

$$noise\_seq = D * A * uniform\_random\_seq,$$

Where D is the noise degree (we use D=0.05 for all datasets), A is the amplitude range of the original signal, $uniform\_random\_seq$ is an independent and identically distributed (i.i.d.) sequence that has the same length as the signal and is generated from a uniform distribution in (-1,1) by $np.random.rand$. To generate the low-frequency noise, we first sample a random noise sequence with $\frac{1}{100}$ of the signal length in the same way, and we later use $scipy.interpolate.interp1d$ to interpolate the noise sequence into the same length, whose frequency will turn low. After generating the noise sequence, we will add the noise to the original signal, where the probability of adding “high” or “low” or “both” will be equal.

Channel Flipping. The sensor on the left side and the right of the brain are placed symmetrically. Thus, we flip the corresponding channels as another augmentation method. For Sleep EDF, we

can flip the Fpz-Cz and Pz-Oz channels; For MGH Sleep, we can flip the F3-M2 and F4-M1, or C3-M2 and C4-M1 or O1-M2 and O2-M1; For SHHS, we can flip C3/A2 and C4/A1.

Shifting. Within one instance, we will rotate/delay the signal for a certain time span. For three datasets, we uniformly split a signal epoch into two pieces and then resemble it as the augmentation. An illustration is provided in Figure 1. After doing augmentations, we will clip the signal amplitudes within a valid sensing range (which is the max measured signal amplitude, 2.5e-4 for Sleep EDF, 50 for MGH, 1.25e-4 for SHHS). To get an augmented version of a signal, we randomly apply one of the augmentation methods with equal probability.

Model Implementation

All models are implemented using PyTorch 1.4.0 and optimized with Adam optimizer, 2e-4 as learning rate, 1e-4 as weight decay. For all datasets, we use 256 as batch size and use $\sigma = 2, \delta = 0.2, T = 2$ as the hyperparameters. In terms of the dimension of learned representations, 128 is for Sleep EDF, 256 for SHHS, and 192 for MGH. We implement the logistic regression model by scikit-learn with default setting and 500 as the maximum iteration for logistic regression evaluation of contrastive methods (the logistic regression is implemented by $klearn.linear\_model.LogisticRegression$ with $max\_iter = 500$ argument.). The experiments are conducted on two NVIDIA GTX 3090 GPUs with 24GB memory each, a 32-core CPU Linux machine with 256GB RAM. Note that the training process is IO-intensive, which involves loading sample files from the disk batch-by-batch. Therefore, a 4TB SSD persistent disk is used to store the raw signal epochs.
