# Supplementary material for: Self-Supervised Electroencephalogram Representation Learning for Automatic Sleep Staging: Model Development and Evaluation Study
Source: JMIR AI. 2023 Jul 26;2:e46769. doi: 10.2196/46769 (PMC10715804; doi:10.2196/46769)
Supplement: Supplementary file 3 [file ai_v2i1e46769_app4.pdf]

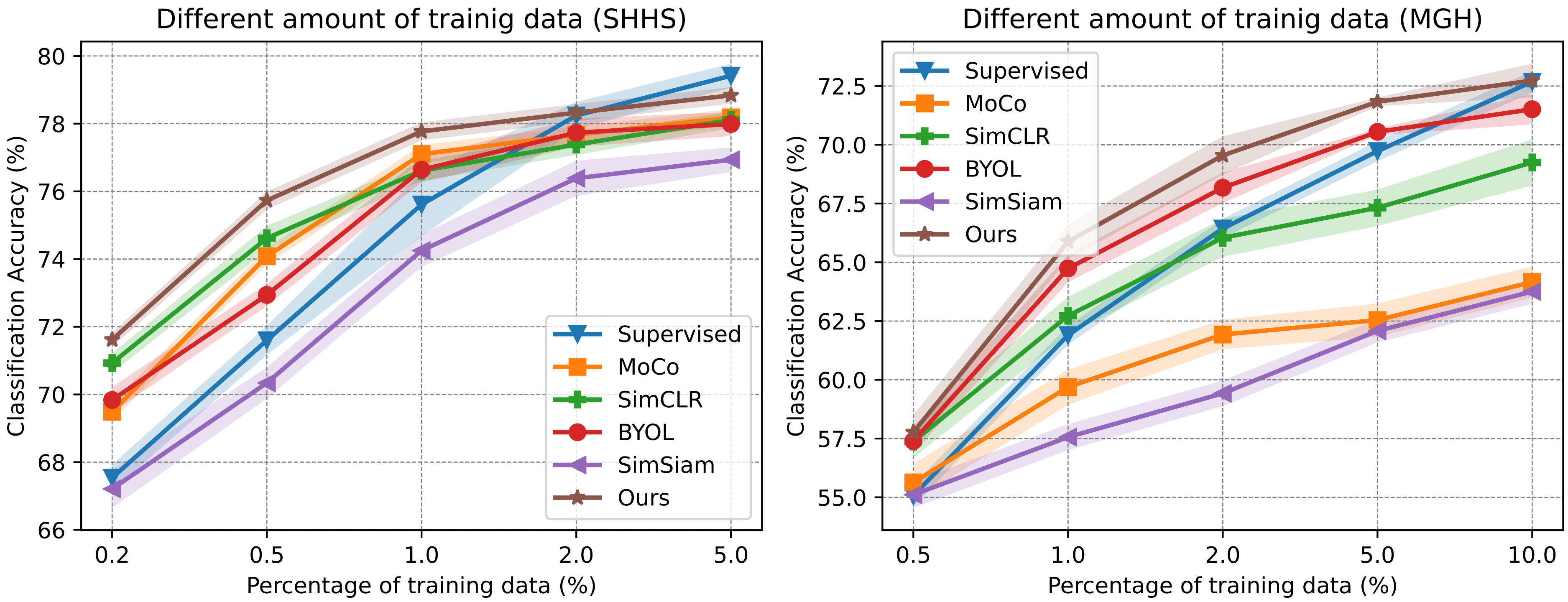

Different amount of trainig data (SHHS)
Different amount of trainig data (MGH)
Classification Accuracy (%)
Percentage of training data (%)
Supervised
MoCo
SimCLR
BYOL
SimSiam
Ours
